# Culturing the desert microbiota

**DOI:** 10.3389/fmicb.2023.1098150

**Published:** 2023-04-11

**Authors:** Zakia Selmani, Eleonore Attard, Béatrice Lauga, Mohamed Barakat, Philippe Ortet, Joris Tulumello, Wafa Achouak, Yahia Kaci, Thierry Heulin

**Affiliations:** ^1^Laboratoire de Biologie et Physiologie des Organismes, Faculté des Sciences Biologiques, University of Science and Technology Houari Boumediene (USTHB), Algiers, Algeria; ^2^CEA, CNRS, BIAM, LEMiRE, Aix-Marseille Université, Saint-Paul-lèz-Durance, France; ^3^E2S UPPA, CNRS, IPREM, Université de Pau et des Pays de l’Adour, Pau, France

**Keywords:** desert, microbiota, diversity, culturability, Sahara

## Abstract

Over the last 30 years, the description of microbial diversity has been mainly based on culture-independent approaches (metabarcoding and metagenomics) allowing an in-depth analysis of microbial diversity that no other approach allows. Bearing in mind that culture-dependent approaches cannot replace culture-independent approaches, we have improved an original method for isolating strains consisting of “culturing” grains of sand directly on Petri dishes (grain-by-grain method). This method allowed to cultivate up to 10% of the bacteria counted on the surface of grains of the three sites studied in the Great Western Erg in Algeria (Timoudi, Béni Abbès, and Taghit), knowing that on average about 10 bacterial cells colonize each grain. The diversity of culturable bacteria (collection of 290 strains) predicted by 16S rRNA gene sequencing revealed that *Arthrobacter subterraneus*, *Arthrobacter tecti*, *Pseudarthrobacter phenanthrenivorans*, *Pseudarthrobacter psychrotolerans*, and *Massilia agri* are the dominant species. The comparison of the culture-dependent and -independent (16S rRNA gene metabarcoding) approaches at the Timoudi site revealed 18 bacterial genera common to both approaches with a relative overestimation of the genera *Arthrobacter*/*Pseudarthrobacter* and *Kocuria*, and a relative underestimation of the genera *Blastococcus* and *Domibacillus* by the bacterial culturing approach. The bacterial isolates will allow further study on the mechanisms of tolerance to desiccation, especially in *Pseudomonadota* (*Proteobacteria*).

## Introduction

Over the last 40 years, three major events have greatly modified our approaches to describing microbial diversity in ecosystems. The first one was in the 1980s with the description of a method for extracting DNA in particular from soils (Torsvik et al., 1990) with numerous improvements to the methods such as environmental DNA (eDNA) (Taberlet et al., 2012). The second was the description of methods for amplifying by PCR this DNA extracted from soils (Steffan and Atlas, 1988; Bruce et al., 1992; Navarro et al., 1992; Stackebrandt et al., 1993), which led to the development of genotyping methods (RFLP and DGGE), 16S rRNA cloning and more recently metabarcoding of 16S rRNA gene and metagenomics. Another important event was the publication of a review by Amann et al. (1995) on the *in situ* hybridization (FISH) for the detection and counting of bacterial populations without culture. In the introduction of this review, the authors cite comparisons between the measurement of the size of “culturable” bacteria (Colony Forming Unit-CFU method) and that made by microscope observations with or without *in situ* hybridization with reference to the “great plate count anomaly” mentioned by Staley and Konopka (1985). The data presented in the Amann’s review highlights that the percentages of culturable bacteria in some terrestrial ecosystems (soil and freshwater) varied between 0.1 and 1%, while in seawater they are below 0.1% (Amann et al., 1995). This article is quoted extensively (7604 in WoS database, March 2023) for this result which, even if it only concerned a few examples of ecosystems, led the many authors citing this article to retain this key value of 1% in the introduction to their own article, justifying the use of culture-independent methods. Molecular methods allow an in-depth analysis of bacterial diversity down to the species level, particularly with the development of high-throughput sequencing methods, which are constantly improving and are becoming less and less costly, allowing for the sequencing of an increasingly large number of samples. In contrast, genotypic and phenotypic microdiversity can only be assessed using the culture-dependent approach. The counterpart of this rapid evolution of techniques for describing molecular bacterial diversity in less than 40 years, is that it has led a generation of young microbiologists who are abandoning the classic approaches of counting and especially isolating bacterial strains, since these culturable populations are only supposed to represent 1% of the real diversity.

The question of the relationship between viability and culturability has been raised for many years (Barer and Harwood, 1999). The low percentage of culturability of environmental bacteria may be due to the fact that some culturable bacteria may enter a temporarily non-culturable state called “viable but non-culturable” (Barer and Harwood, 1999) and to the difficulty of finding the adequate medium to allow the growth of bacteria (culturability *sensu stricto*), but also to the method of separating the bacteria from the solid phase on which they are attached (extractability). Many mechanisms are involved in the adhesion of bacteria to surfaces and can lead to the irreversibility of this adhesion (Berne et al., 2018). This is particularly true in soils as the vast majority of bacteria are associated with aggregates of varying size that are very difficult to dissociate in the suspension-dilution steps prior to plating on Petri dishes.

In a previous publication, on sand samples from the Merzouga dune (Morocco), considering the extractability challenge, we evidenced that a method of depositing sand grains individually and directly on Petri dishes (the so-called “grain-by-grain” method) allowed bacterial colonies to grow around the sand grains, without requiring any extraction step (Gommeaux et al., 2010). With this method, the percentage of culturable bacterial cells was 14% (% of bacterial cells counted by fluorescence under the microscope), compared with the conventional suspension-dilution method (<2%) (Gommeaux et al., 2010).

In this article, we undertook a more ambitious study (three sites more than 100 km apart) in the dunes of the Great Western Erg of the Sahara (Algeria) to assess the intra- and inter-sites variability of bacterial biodiversity using two methods [suspension-dilution (SD) and grain-by-grain (GbG)], to estimate the percentage of bacterial culturability, to build up a collection of strains for which the identification was predicted at the species level, and finally to compare this diversity of culturable bacteria with that obtained by 16S rRNA gene metabarcoding.

## Materials and methods

### Field location and sampling

Soil samples were collected from non-vegetated sand dunes in three sites in Southern Algeria (from South to North: Timoudi/TM, Béni Abbès/BA, and Taghit/TG). GPS coordinates and altitudes are detailed in Supplementary Table 1. The climate is arid, with an average annual rainfall of about 87 mm (falling over less than eight rainy days per year). From May to August, the region experiences a particularly dry summer with daytime temperatures often above 37°C, with nights being approximately at 23.5°C. Soil samples were collected from 9 to 11 September 2014, at a depth of 0–5 cm, placed in sterile airtight disposable plastic tubes (approximately 50 g) and bags (approximately 250 g). For each site, three sub-sites were sampled (TM1/TM2/TM3, BA1/BA2/BA3, and TG1/TG2/TG3), and for each sub-site, three replicates were sampled (except for TG2 and TG3, two replicates). The average distance between sub-sites was 100–200 m (in a triangular shape) and 5 m between replicates (in a triangular shape). Samples were analyzed between September 2014 and July 2015 (physicochemical and microbiological analyses). DNA extraction was performed in March 2017.

### Mineralogical analysis

The physicochemical characterization of sand samples from the three sites (TM, BA, and TG) focused on mineralogical composition using energy-dispersive spectroscopy (EDS).

### Enumeration of grains per gram of sand

To determine the number of grains per gram of sand, we first weighed a 10 mg sample of sand for the different sub-sites (TM1/TM2/TM3, BA1/BA2/BA3, and TG1/TG2/TG3), with three replicates per sub-site. We then counted the number of grains for each sample using a magnifying glass, and then expressed it per gram of sand.

### Granulometry and mineralogical analyses

The determination of the sand grain size was carried out at the Laboratory of Sciences and Engineering of Materials (Houari Boumediene University, Algiers, Algeria) on a Malvern laser granulometer (Mastersizer 2000 Malvern Instruments). The sizes ranged from 50 nm to 1,000 μm. Concentrated suspensions (1.0% m:v), were prepared using appropriate wetting and/or dispersing agents.

### Enumeration of the total number of bacteria

The fluorescent dye Syto 9 (Molecular Probes) was used according to the manufacturer’s instructions. Staining of 0.1 g sand samples was performed and a series of 160 bulk sand grains were observed using a low magnification light microscope, typically ×100, after mounting in CoverWell imaging chambers (Schleicher and Schuell). The epifluorescence system consisted in a burner and a pair of filters corresponding to the excitation and emission wavelength of the Syto 9 dye (480 nm for excitation and 500 nm for emission). Over 100 grains were analyzed per site (TM:131, BA:120, and TG:107). Only the upper half of the grain was accessible with this method. It was assumed that the number of fluorescence spots on the lower half of the grain was the same as on the upper half, so the number of fluorescence spots was doubled. The number per grain was converted to number per gram by estimating the number of sand grains per gram.

### Enumeration and isolation of culturable bacteria using suspension-dilution technique

The sand samples (1 g per sample) were manually ground in a sterile mortar with 1 ml sterile water for 3 min. An additional sonication treatment was performed, using an ultrasonic homogenizer (VibraCell, Bioblock Scientific) for 10 s. The supernatant was removed and serially diluted to 10^–2^ in KCl 0.9 g/L. Dilutions were spread on 10-fold diluted Tryptic Soy Agar solid medium (TSA 1/10) (Difco Laboratories). After incubation at 30°C for 15 days, plates containing 20–50 colonies were used for enumeration. For the isolation step, all colonies of the highest dilution on Petri dishes were used without any morphological selection, and purified by streaking on the same medium. Two independent experiments were performed.

### Enumeration and isolation of culturable bacteria using grain-by-grain method

Grain-by-grain method was conducted on TSA 1/10 according to the protocol described by Gommeaux et al. (2010). Seven individual grains were picked per Petri dish with a drop (50 μl) of TSB 1/10 per grain. Petri dishes were incubated at 30°C. Counting was performed after 15 days of incubation and all colonies were isolated and purified on TSA 1/10. As only the lower hemisphere of the sand grains was in contact with the surface of the agar nutrient medium, the number of colonies counted was doubled. Two independent experiments were performed.

### Molecular identification of bacterial strains

Bacterial strains were grown in TSB 1/10 until turbidity. Samples were centrifuged at 9,600 *g* for 3 min to retrieve cells and the following thermic protocol was applied to DNA extraction: 95°C for 5 min, 4°C for 3 min, 95°C for 5 min and 4°C for 3 min. The 16S rRNA genes were amplified using the universal primers fD1 (5′-AGAGTTTGATCCTGGCTCAG-3′) (Weisburg et al., 1991) and S17 (5′-CGGTCACGTTCGTTGC-3′) (Ruimy et al., 1994). The reaction mixture (50 μl final volume) contained 10 μl of GoTaq^®^ Flexi Buffer^1^, 3 μl (1.5 mM) MgCl_2_ solution, 2 μl (200 μM) dNTP, 2 μl (1 μM) of each primer, 0.25 μl (1.25 u) GoTaq^®^G2 Flexi DNA Polymerase (5 u μl^–1^) and 2 μl of template DNA. PCR amplification consisted of an initial denaturation at 95°C for 2 min, followed by 34 cycles of denaturation at 95°C for 30 s, annealing at 53°C for 30 s, extension at 72°C for 90 s and a final extension at 72°C for 5 min. PCR products were sequenced using standard Sanger sequencing technique (GENEWIZ, Beckman). The complete or partial 16S rRNA sequences of the bacteria were blasted within the National Center for Biotechnology Information server.^1^ The 16S rRNA gene sequences of 439 strains were compared with the most similar neighboring phylogenetic sequence(s) from GenBank databases with RefSeq accession numbers ON629815 to ON630253.^2^ We completed the identification prediction using a second databases, EzBioCloud.^3^ The length of the 16S rRNA gene sequences we obtained was variable, due to the difficulty of sequencing some PCR products despite the use of four primers: fd1, S10 (5′-CCGTCAATTCATTTGAGTTT-3′), P3 (5′-GCCTACGGGAGGCAGCAG-3′), and S17. Therefore, bacterial strains were identified using the whole 16S rRNA gene or its V1–V4 region and assigned to species when the percentage of identity was higher than 98.5% (whole 16S rRNA gene) or 98.9% (V1–V4 region), which corresponds to the threshold proposed by Meier-Kolthoff et al. (2013).

### Bacterial diversity of sand grain using culture-independent method

DNA was extracted from 10 g of sand using the DNeasy PowerMax Soil Kit (Qiagen, France) according to the manufacturer’s instructions. The DNA extracts were then concentrated 50-fold *via* an ethanol precipitation step. The V4–V5 hypervariable region of the 16S rRNA gene targeting Bacteria and Archaea was amplified using the primers 515F (5′-GTGYCAGCMGCCGCGGTA-3′) and 928R (5′-CCGYCAATTCMTTTRAG-3′). The reaction mixture included (final volume/concentration) 0.5 μM of each primer, 1X of Q5 High-Fidelity 2X Master Mix (New England Biolabs, Evry, France), 0.5 μg of T4 gene 32 protein (New England Biolabs, Evry, France) and 2.5 μl of template DNA. Cycling conditions included an initial denaturation at 98°C for 30 s, followed by 30 cycles of denaturation at 98°C for 10 s, annealing at 70°C for 30 s and extension at 72°C for 30 s and an additional extension step at 72°C for 2 min after cycling was complete. Triplicates PCR reactions were performed for each sample and concentrated by ethanol precipitation to the volume required for the sequencing protocol. Illumina MiSeq sequencing was performed using the 2 × 250 paired-end protocol with an Illumina^®^ MiSeq instrument at the GeT plage facility, Toulouse, France.^4^

The analysis was performed using FROGS, a Galaxy-supported pipeline designed to consider large sets of amplicon sequences and produce abundance tables of Operational Taxonomic Units (OTUs) and their taxonomic affiliation (Escudié et al., 2018). The clustering was achieved using Swarm (Mahé et al., 2014). Metabarcoding SRA data are available with the BioProject accession number PRJNA837115.^5^

### Bioinformatics and statistical analyses

Statistical tests and graphical representation were performed using R software version 3.5.3. Data processing and graphing were done using *tidyverse* packages (Wickham et al., 2019), correlation between variables were performed using *cor* function. For each pairwise test, *p*-values were adjusted by BH correction. Each univariate analysis was performed using the Kruskal--Wallis test. If a parameter had a significant influence on a variable, a pairwise *post-hoc* Dunn test was used to determine group significance differences using *PMCMRplus* package.^6^ Homogeneity groups were calculated automatically using the *multcompLetters* function with a *p-*value threshold = 0.05. Alpha-diversity was calculated by taxonomic richness (i.e., the number of taxa, expressed as the number of observed OTUs) and Chao1 index. Shannon index was used then for evenness estimation between samples.

Heatmap analyses of microbial community structure, coupled to a hierarchical clustering, were performed based on Bray-Curtis dissimilarity matrices using the heatmap.2 function from the *gplots* package^7^ and the *hclust* R function. Dissimilarity indices were calculated using the function *vegdist* of the vegan package.^8^

## Results

**FIGURE 1:**
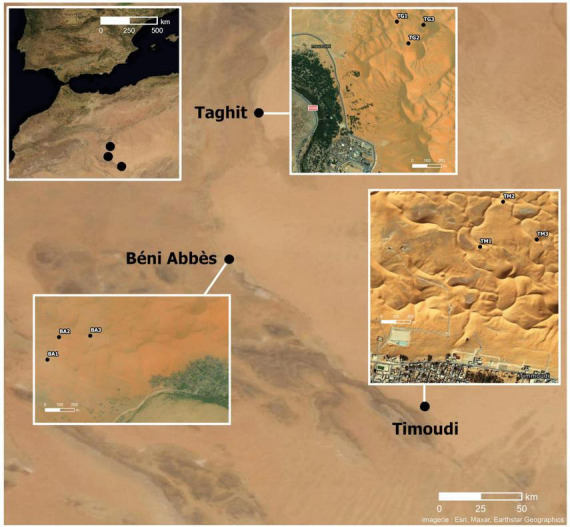
Location of the three sites at the edge of the Grand Erg Occidental in Algeria: from South to North: Timoudi, Béni Abbès, and Taghit. Distance between Timoudi and Béni Abbès: 150 km and between Taghit and Béni Abbès: 100 km.

The three sites, from South to North, Timoudi/TM, Béni Abbès/BA, and Taghit/TG, are located in southern Algeria on the edge of the Grand Erg Occidental (Figure 1 and Supplementary Table 1).

### Grain mineralogy, grain counting, and average grain diameter

The sand grains at all three sites (TM, BA, and TG) consisted mainly of silicates (Si and O), other silicates such as feldspars and micas (Si, O, Al, and Na/K), and some calcite grains (Ca and O). No differences were observed between the three sites in terms of mineralogy.

**FIGURE 2:**
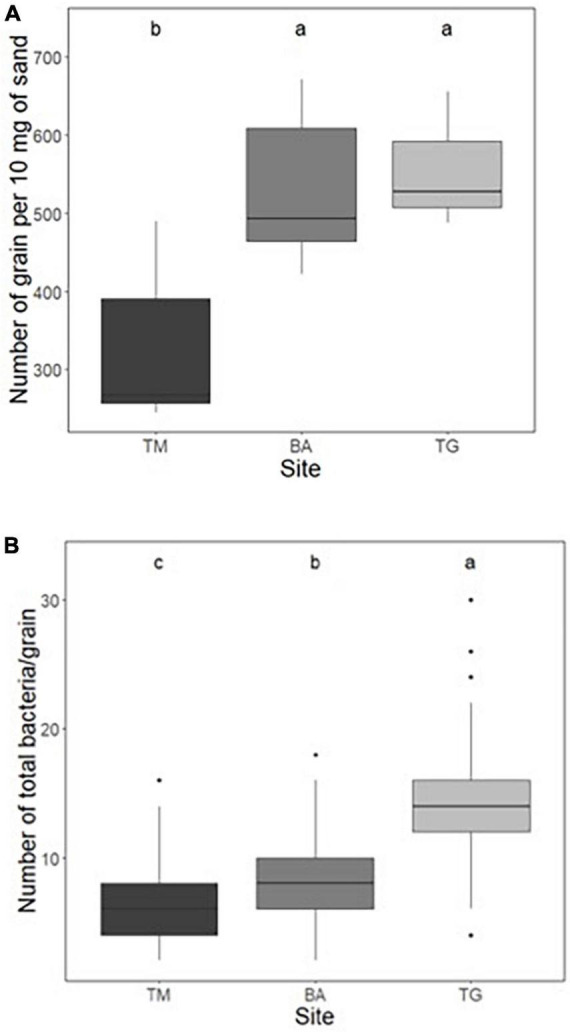
**(A)** Number of grains per 10 mg of sand measured by manual counting and **(B)** number of total bacteria per grain by microscope observations (after Syto9 staining) at the three sites: Timoudi (TM), Béni Abbès (BA), and Taghit (TG). Letters represent homogeneity groups based on Kruskal–Wallis *p-*values adjusted with BH correction using a 0.05 threshold.

The number of grains per gram of sand was first counted by hand on 10 mg of sand for each sub-site. The analysis of variance (ANOVA) showed a highly significant effect of the location on this parameter, ranging from 325 ± 88 (TM), 501 ± 85 (TG) to 531 ± 95 (BA) grains per 10 mg sand (Figure 2A and Supplementary Table 2). These figures were confirmed by the use of the granulometer: from 32125 (TM), 47771 (TG) to 51325 (BA) grains per g (Supplementary Table 3). Regardless of how the average diameter of sand grains was calculated (using the weighted surface D[3,2] or weighted volume D[4,3]), the grains at the TM site were the largest and those at the BA site the smallest (Supplementary Table 3), in agreement with the ranking of the number of grains per gram of sand (TM < BA = TG). Using the mean diameter estimated from the weighted volume D[4,3], the mean surface and volume of sand grains at the TM site (0.38 mm^2^, 0.022 mm^3^) were larger (+50 and +70%, respectively) than those at the BA site (0.26 mm^2^, 0.013 mm^3^) (Supplementary Table 3).

### Counting total bacteria per grain

Using fluorescence imaging to count the number of total bacteria per grain, we found that the population size was significantly different (*p* < 0.01, Kruskal–Wallis test) between the three sites, and ranged from 6.6 ± 2.3 (TM), 8.1 ± 2.9 (BA) to 14.3 ± 4.5 (TG) (Figure 2B and Supplementary Table 4), representing from 2.1 (TM) to 7.1 × 10^5^ total bacteria.g^–1^ of sand at the TG site.

### Counting culturable bacteria per grain: Grain-by-grain method

**FIGURE 3:**
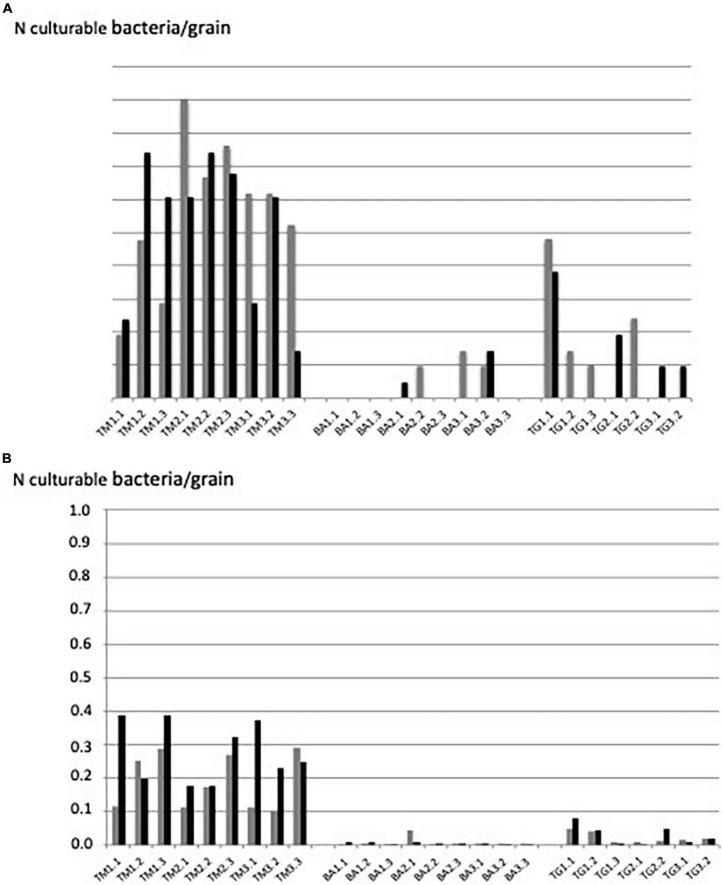
Number of culturable bacteria per grain revealed by **(A)** “grain-by-grain” method and **(B)** “suspension-dilution” method at the three sites: Timoudi (TM), Béni Abbès (BA), and Taghit (TG) and two independent experiments (Exp1 in gray and Exp2 in black).

All TM sand grains were colonized by 0.1–0.9 bacterial cells per grain in average (1–9 bacterial cells per 10 grains) in the two independent experiments (Figure 3A). In contrast, less than 30% of the grains of BA were colonized by a few culturable bacterial cells (about 0.1 bacteria per grain). The picture of TG grains was intermediary (about 50% of grains colonized by 0.03–0.54 bacteria per grain) (Figure 3A), representing between 0.1 and 1.7 10^4^ culturable bacteria.g^–1^ sand (BA and TM, respectively). Reproducibility between the two independent experiments was highly significant (*p* < 0.01; see correlation curve in Supplementary Figure 1).

### Counting culturable bacteria per grain: Suspension-dilution method

The same ranking of the number of culturable bacteria per grain was obtained using the conventional “suspension-dilution” method, but with a lower number of bacteria per grain regardless the site: from less than 0.1 bacteria per grain for the BA site to 0.1–0.4 bacteria per grain for the TM site (Figure 3B). The reproducibility between the two independent experiments was highly significant (*p* < 0.01; see correlation curve in Supplementary Figure 2).

### Correlation between culturable bacteria per grain using GbG and SD methods

To assess the correlation between the two methods, we first expressed the number of culturable bacteria per grain as a percentage of the total bacteria per grain. This correlation was highly significant (*p* < 0.01) (Supplementary Figure 3). On average, this percentage was twice as high when using the GbG method compared to the SD method (y = 1.85x). For several TM samples, the percentage of culturable bacteria obtained with the GbG method was higher than 10% compared to total number of bacteria. If all of the fluorescent signals were not associated to live bacteria, this means that the percentage of culturability could be higher than 10%.

### Diversity of culturable bacteria

**FIGURE 4:**
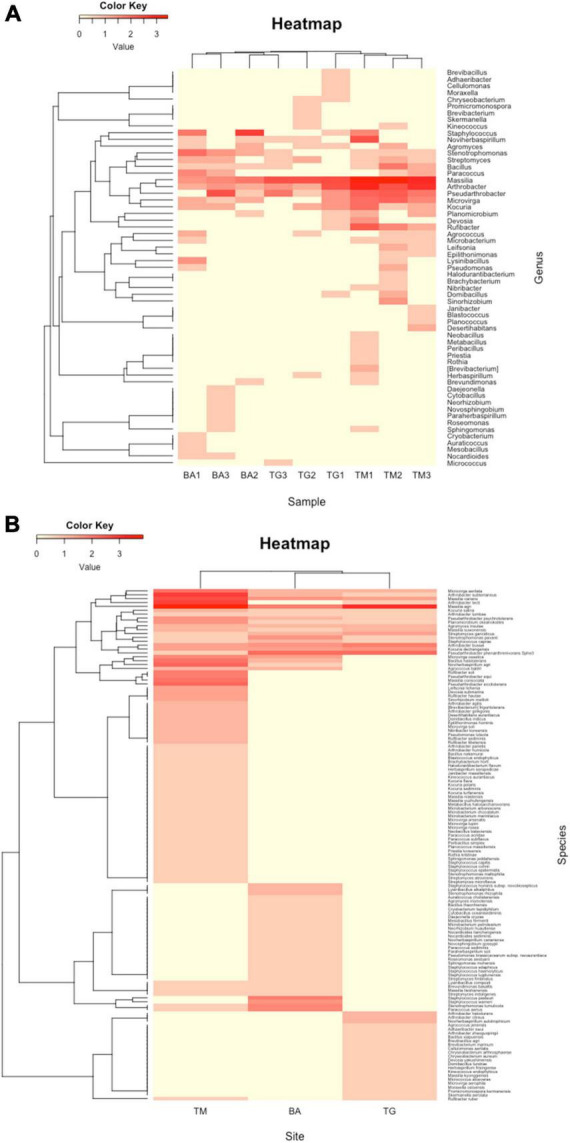
Representation of clustering in the form of heatmap of **(A)** the complete list of strains isolated and **(B)** the list of strains predicted at the species level at the three sites: Timoudi (TM), Béni Abbès (BA), and Taghit (TG).

A total of 507 bacterial strains were isolated from the 3 sites from which 439 were identified at the genus or species level (87%), including 251 strains isolated from Timoudi (103/87/61, respectively in TM1/TM2/TM3; Supplementary Table 5A), 103 strains isolated from Béni Abbès (40/29/34, respectively in BA1/BA2/BA3; Supplementary Table 5B), and 85 strains isolated from Taghit (39/24/22, respectively in TG1/TG2/TG3; Supplementary Table 5C). The identification prediction was performed using both NCBI NR and EzBioCloud databases. Of the 439 bacterial strains, 290 strains were identified to species level (66%) (Supplementary Table 6), and the remainder to genus level (19%) or potential new species with identity percentage of partial 16S rRNA sequence lower than 98.8 (15%). As expected, we found that the variability in culturable bacterial diversity between sites (inter-sites) was greater than that within sites (intra-site), with greater homogeneity of BA samples compared to the other two sites (Figure 4A). Regardless of the methods used to assess richness (Chao1, observed species/OTUs: Operational Taxonomic Units) and evenness (Shannon), alpha-diversity was significantly higher at the TM site than at the BA and TG sites (*p* < 0.05) (Supplementary Table 7). The richness estimate was 31 ± 5 OTUs at the TM site compared to 16 ± 6 OTUs at the BA and TG sites. All main data presented above have been summarized in Table 1.

**TABLE 1:** Data synthesis of total bacteria, culturable bacteria per g of sand, and number of OTUs at the three sites.

	Sites		
	Timoudi	Béni Abbès	Taghit
N grains.g^–1^ sand	3.2^b^ + 0.9 10^4^	5.3^a^ ± 0.9 10^4^	5.0^a^ ± 0.8 10^4^
Mean grain surface (mm^2^)	0.38	0.26	0.30
Grain volume (mm^3^)	0.022	0.013	0.016
Total bacteria/grain	6.6^c^ ± 2.3	8.1^b^ ± 2.9	14.3^a^ ± 4.5
Total bacteria.g^–1^ sand	2.1^c^ ± 0.7 10^5^	4.3^b^ ± 1.1 10^5^	7.1^a^ ± 1.1 10^5^
Culturable bacteria/grain (grain-by-grain)	0.54^a^ ± 0.22	0.03^b^ ± 0.05	0.12^b^ ± 0.15
Culturable bacteria.g^–1^ sand (grain-by-grain)	1.7^a^ ± 0.6 10^4^	0.1^b^ ± 1.8 10^4^	0.6^b^ ± 0.7 10^4^
OTUs	31^a^ ± 5	16^b^ ± 6	16^b^ ± 3

Mean ± SD. Means with different letters are significantly different (*p* < 0.05).

Considering the prediction of the identification at genus level, hierarchical clustering revealed that the three BA replicates were different from those of TM and TG (Figure 4A). This difference was mainly due to a higher percentage of the genera *Staphylococcus* and *Stenotrophomonas* (Supplementary Table 8) in the BA samples compared to the other two. Conversely, the percentage of the genera *Arthrobacter/Pseudarthrobacter* and *Massilia* was lower at the BA site compared to the other two (Figure 4A and Supplementary Table 8).

We analyzed the reduced list of 290 strains predicted at the species level (Supplementary Table 6), eliminating strains identified as “*genus*. sp.” and as “*genus.* sp. nov.” In the corresponding heatmap, the diversity at the TM site was clearly different compared from that at the BA and TG sites (Figure 4B). Exploring the bacterial species explaining the difference between the diversity expressed at genus and species level, we evidenced that *Arthrobacter subterraneus* and *Arthrobacter tecti* were more frequent at the TM site whereas at the BA and TG sites *Pseudarthrobacter phenanthrenivorans* (formerly *Arthrobacter phenanthrenivorans*) was the most frequent (Supplementary Table 9). We also found that *Massilia agri* was the most frequent at the BA and TG sites, whereas at the TM site *Massilia* sp. was the most frequent (Supplementary Table 9). In addition to these differences within *Arthrobacter/Pseudarthrobacter* and *Massilia* genera, the TM site was characterized by a list of 35 bacterial species that were absent at the BA and TG sites (Figure 4B).

### Bacterial diversity using 16S rRNA metabarcoding

**FIGURE 5:**
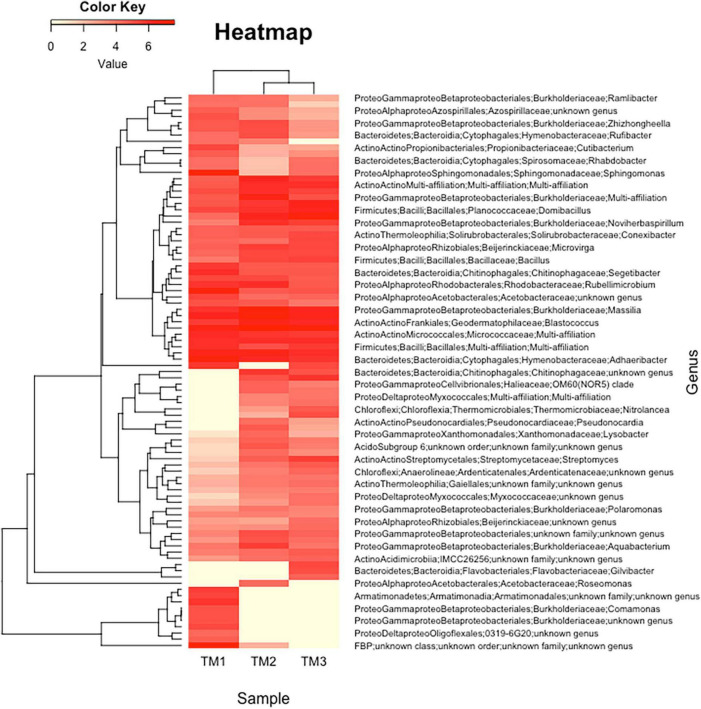
Representation of clustering in the form of heatmap of the complete list of strains predicted using 16S rRNA gene metabarcoding in Timoudi (TM) site, highlighting the bacterial genera differentiating the three sub-sites of TM.

The objective was to describe the culture-independent diversity using 16S rRNA gene metabarcoding of the three sites. We were only able to extract sufficient DNA from the TM site (three sub-sites), probably because the number of culturable bacteria per g of sand was the highest in this site (Table 1). In total, 175 OTUs were identified in the three TM sub-sites, after removal of singletons (Supplementary Table 10). The diversity of the three TM sub-sites is presented in the form of heatmap (Figure 5). The most frequent OTU was represented by an unknown genus belonging to Frankiales (8.3%), followed by genera belonging to *Bacteroidota* (*Bacteroidetes*) (*Flavisolibacter* 5.5%, *Adhaeribacter* 4.2%, *Nibribacter* 4.0%), and other Actinomycetota (*Actinobacteria*) (*Nocardioides* 3.8%, *Blastococcus* 3.3%, *Geodermatophilus* 2.7%). In total, the genera belonging to *Actinomycetota* (*Actinobacteria*) and *Bacteroidota* accounted for 25 and 20%, respectively. The most frequent *Pseudomonadota* (*Proteobacteria*) were *Massilia* (4.7%) and *Sphingomonas* (2.9%) (Supplementary Table 10). Among the rare genera revealed by using metabarcoding, we found *Ramlibacter* (0.3%) (Figure 5, first line).

### Comparison of culture-dependent and independent approaches

**FIGURE 6:**
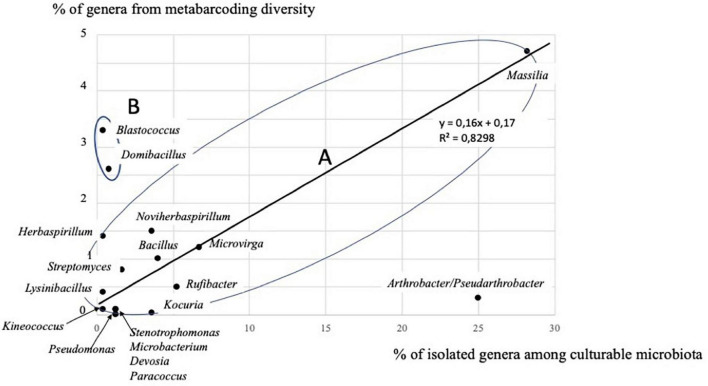
Correlation between the two approaches (culture-dependent and -independent) considering 15 genera in group A, out of the 18 genera detected by both approaches at the Timoudi site (see Supplementary Table 11).

The comparison of the diversity of isolated strains and 16S rRNA metabarcoding showed a fairly good convergence in the identification of the dominant bacterial genera, as 18 genera were found with both approaches (Supplementary Table 11). These 18 genera represented 85.9% of all genera in the culturable microbiota, and 18.8% of the genera detected using 16S rRNA metabarcoding (Supplementary Table 11). The genus *Massilia* (*Betapseudomonadota*-*Betaproteobacteria*) was found to be dominant in culturable microbiota and also using 16S rRNA gene metabarcoding (28.2 and 4.7%, respectively). Other dominant genera in culturable microbiota were present using culture-independent approach, such as *Arthrobacter/Pseudarthrobacter* (25.0 vs. 0.3%, respectively), *Rufibacter* (5.2 vs. 0.5%), *Microvirga* (6.7 vs. 1.2%), *Bacillus* (4.0 vs. 1.0%), *Kocuria* (3.6 vs. 0.04%), and *Noviherbaspirillum* (3.2 vs. 1.5%). Dominant genera detected using metabarcoding, other than *Massilia*, were also present in culturable microbiota, e.g., *Blastococcus* (3.3 vs. 0.4%), and *Domibacillus* (2.6 vs. 0.8%). If we consider the 18 dominant genera of the culturable microbiota (Supplementary Table 11), there is a significant correlation (*p* < 0.01) between the two approaches (culture-dependent and -independent) within the group A (Figure 6), excluding *Blastococcus*, *Domibacillus*, and *Arthrobacter*/*Pseudarthrobacter* genera.

Surprisingly, another 20 genera found in culturable microbiota were absent using 16S rRNA gene metabarcoding (Supplementary Table 11). Compared to culture-independent approach, the culture-dependent approach tends to overestimate the genera *Arthrobacter/Pseudarthrobacter* and *Kocuria* and to underestimate the genera *Blastococcus*, and *Domibacillus* (group B; Figure 6 and Supplementary Table 11). Both approaches confirm the dominance of *Massilia* strains (group A; Figure 6 and Supplementary Table 11). More generally, the culture-dependent approach overestimated *Betapseudomonadota* (28.6 vs. 6.1% with 16S rRNA gene metabarcoding) and underestimated *Bacteroidota* (7.6 vs. 19.5% with metabarcoding) and was more or less close for *Actinomycetota* (*Actinobacteria*) (38.6 vs. 25.0% with 16S rRNA gene metabarcoding), *Bacillota* (*Firmicutes*) (10.4 vs. 5.9% with metabarcoding) and *Alphapseudomonadota* (*Alphaproteobacteria*) (10.7 vs. 7.2% with 16S rRNA gene metabarcoding) (Supplementary Table 11).

## Discussion and conclusion

No differences were observed between the three sites (TM, BA, and TG) in terms of mineralogy. The grains are mainly silicates, feldspars, micas, and some calcite grains, in all respects comparable to the mineralogical diversity found in Merzouga (Morocco), which is at the end of the Grand Erg Occidental (Gommeaux et al., 2010).

The southernmost site (TM) was characterized by a larger grain diameter (+13%) compared to the other two sites (BA and TG), which resulted in a significantly lower number of grains.g^–1^ (−40% on average/BA and TG) (Table 1). Although the opposite might have been expected, the total number of bacteria per grain at the TM site, counted directly under the microscope, was significantly lower than at the other two sites (−20 to −50%), resulting in a significantly lower total number of bacteria.g^–1^ (−50 to −70%) (Table 1). On the other hand, the estimation of the number of culturable bacteria.g^–1^, using the “grain-by-grain” method, showed that the number of culturable bacteria at the TM site was significantly higher than at the other two sites (>100%), this culturable microbiota being twice as diverse as at the BA and TG sites (Table 1). In summary, the sand grains at the TM site are significantly larger than those at the other two sites (BA and TG) and were colonized by a larger culturable microbiota (1.7 × 10^4^ bacteria.g^–1^ sand at the TM site compared to <0.6 × 10^4^ bacteria.g^–1^ sand at the BA and TG sites) and more diverse (31 OTUs compared to 16 OTUs), despite the smaller total microbiota size. The size of these bacterial populations is one log larger than that of the Barchan Qatari sand dunes (2.2 × 10^3^ bacteria.g^–1^) given by Abdul Majid et al. (2016) and close to that of a Saharian site, Merzouga (2.2 × 10^3^ bacteria.g^–1^) published by Gommeaux et al. (2010).

Several hypotheses can be considered, in an attempt to explain the apparent contradiction observed between the total number of bacteria per grain (TM < BA < TG) and the number of culturable bacteria per grain (TM > BA = TG) (Table 1). The first hypothesis is that the larger grains at the TM site (TM > BA = TG) (Table 1) are associated with larger inter-grain pores (mostly air-filled) and therefore aerobic conditions compared to the smaller grains at the BA and TG sites, characterized by smaller pores and potentially under aerophilic to microaerophilic conditions, which are more favorable to bacterial growth on the grain surface (TM < BA < TG) (Table 1). The second hypothesis is independent of the first one and is based on the GbG culture method. With larger and heavier grains deposited on the surface of the nutrient agar, a greater number of bacterial colonies capable of growth is expected. This is the case for the grains from the TM site (mean grain surface +50% compared to BA site) which could explain the higher number of culturable bacteria per grain (TM > BA = TG) (Table 1).

In this work, we confirmed that the “grain-by-grain” method was the most appropriate to describe the culturable microbiota, since on average, on the three sites, the size of the culturable microbiota was twice that obtained with the conventional “suspension-dilution” method, allowing in the case of the TM site this allowed to describe up to 10–15% of culturable bacteria in an extreme environment (sand grains on the surface of the dunes), confirming previous results (Gommeaux et al., 2010), and well above the 1% usually claimed in most of publications, for instance with sand samples from the hyper-arid Qatari desert (Abdul Majid et al., 2016). To date, this percentage of culturable bacteria has never been achieved in an extreme environment such as deserts.

We found that the dominant culturable genera were *Massilia* (5 species) and *Arthrobacter/Pseudarthrobacter* (17 species) at all three sites (Supplementary Tables 8, 9). We isolated 104 strains of *Arthrobacter/Pseudarthrobacter*, representing 30% of the culturable microbiota on average at the three sites (Supplementary Table 8). The most frequent species (Supplementary Table 9) were *A. subterraneus*, whose type strain was isolated from the deep subsurface waters (Chang et al., 2007), *A. tecti* (type strain isolated from mural paintings) (Heyrman et al., 2005) and *P. phenanthrenivorans* (type strain isolated from creosote-contaminated soil) (Kallimanis et al., 2009). Three species of *Arthrobacter* have recently been described from desert soils, *Arthrobacter liuii* isolated from Xinjiang desert soil, China (Yu et al., 2015), *Arthrobacter deserti* isolated from Turpan desert soil, China (Hu et al., 2016), and *Arthrobacter mobilis* isolated from Cholistan desert soil, Pakistan (Ye et al., 2020), all of which were absent in the three Algerian sites. The presence of *Arthrobacter* species has also been evidenced in the Merzouga dune (Morocco) using culture-dependent and -independent approaches (Gommeaux et al., 2010). In contrast to the genus *Arthrobacter*, which has been frequently identified in desert soils, the more recently described genus *Massilia* (La Scola et al., 1998) has been rarely described in these environments. Two species have been described from desert environment: *Massilia armeniaca* isolated from desert soil in Inner Mongolia, China (Ren et al., 2018) and *Massilia arenae* isolated from a sandy soil in the Qinghai-Tibetan Plateau, China (Zhang et al., 2020). The two species found in our work, *M. agri* and *Massilia varians* (Supplementary Table 6), had never been described before in desert soils.

Surprisingly, only 11 strains of *Streptomyces* were isolated from all three sites (Supplementary Table 6), representing 2–4% of the culturable microbiota (Supplementary Table 8). At the TM site, there was a good match between the estimated culturable *Streptomyces* population (1.6%) and that found using the culture-independent approach (0.8%) (Supplementary Table 11), in contrast with data from Gurbantunggut Desert (Li et al., 2021) showing a very low percentage of *Streptomyces* (<0.4%) using 16S rRNA metabarcoding compared to culture-dependent method (up to 40%). Actinomycetes have been described for a very long time in deserts (Diab and Al Zaidan, 1976) and, from 2000 to 2021, 129 new *Streptomyces* species were reported from 35 deserts worldwide (Xie and Pathom-Aree, 2021), e.g., *Streptomyces atacamensis* and *Streptomyces deserti* isolated from Atacama Desert soil (Santhanam et al., 2012a,b). We found five *Streptomyces* species in the three Algerian sites, *Streptomyces atrovirens, Streptomyces gancidicus, Streptomyces indoligenes, Streptomyces microflavus*, and *Streptomyces fimbriatus* (Supplementary Table 6), never isolated from desert soils before.

We also found five species of *Kocuria*: *Kocuria dechangensis*, *Kocuria turfanensis*, *Kocuria salina*, *Kocuria sediminis* and *Kocuria flava* (Supplementary Table 6), but not the only one previously described in desert soil, *Kocuria aegyptia* (Li et al., 2006). Similarly, two species of *Pseudomonas*, *Pseudomonas luteola* and *Pseudomonas brassicacearum* were isolated (Supplementary Table 6), but not the species previously described in desert soil, *Pseudomonas arsenicoxydans* (Campos et al., 2010), *Pseudomonas duriflava* (Liu et al., 2008) and *Pseudomonas xinjiangensis* (Liu et al., 2009).

The culture-independent approach relatively underestimated the proportion of *Arthrobacter* and failed to identify *Kocuria* (Supplementary Table 11 and Figure 6) suggesting that the culture-dependent approach allowed isolation of rare species as has been reported in the marine environment (Demko et al., 2021). We found very few sequences of Cyanobacteria (mainly *Aliterella* and an unknown genus of Sericytochromatia) at the Timoudi site using metabarcoding (Supplementary Table 10), in agreement with data showing that, in hyperarid deserts, Cyanobacteria are unevenly distributed, only abundant in some biocrusts (Bay et al., 2021), and restricted generally to protected sublithic niches (Makhalanyane et al., 2013, 2015). We cannot exclude PCR amplification problems to explain why we found very few Archaea sequences (only some Euryarchaeota) in contradiction with literature data (Chanal et al., 2006; Fierer et al., 2012).

In total, we predicted the identification of 290 bacterial strains at the species level and probably isolated more than 95 bacterial species from the three sites (63/TM + 19/BA absents at the TM site, +13/TG absents at the TM and BA sites), considering potential additional new species to be identified, including species for which the desiccation tolerance is well-known such as production of bacterial endospores by *Bacillota* and exospores by *Actinomycetota*. Some species of Pseudomonadota evidenced by 16S rRNA metabarcoding were also described for their ability to face desiccation through the formation of cysts such as *Ramlibacter*, confirming earlier results obtained in Tunisian desert (Heulin et al., 2003; Chanal et al., 2006), or the ability of *Deinococcus* to repair DNA damage (de Groot et al., 2005; Chanal et al., 2006).

This work confirms the importance of in-depth analysis of this biodiversity by 16S rRNA gene metabarcoding to obtain a more complete description. The interest of characterizing the diversity of the culturable microbiota is that it is not only complementary to the 16S rRNA gene metabarcoding approach (detection of certain genera only with the culture-dependent approach) but also the only one to provide identifications at the species level and finally to have bacterial strains on which physiology studies are possible. This last point is particularly important for identifying mechanisms that allow *Pseudomonadota* to tolerate desiccation, as for example the recently described in *Ramlibacter tataouinensis* for which it has been shown that bacteriophytochromes play an important role in adaptation to water scarcity (De Luca et al., 2019).

## Data availability statement

The datasets presented in this study can be found in online repositories. The names of the repository/repositories and accession number(s) can be found in the article/Supplementary material.

## Author contributions

YK and TH designed the research, carried out the sampling, and supervised this study. ZS carried out the sample processing and wrote the first draft. WA, TH, and YK provided the experimental materials. ZS, EA, and BL performed the laboratory work. MB and PO conducted the bioinformatics data analysis. JT conducted the statistical analyses. All authors read, reviewed, and edited previous versions of the manuscript and approved the submission.

## Footnotes

2
https://www.ncbi.nlm.nih.gov/nuccore/?term=ON629815:ON630253%5baccn%5d

3
https://www.ezbiocloud.net/

4
http://get.genotoul.fr

5
https://www.ncbi.nlm.nih.gov/sra/PRJNA837115

6
https://CRAN.R-project.org/package=PMCMRplus

7
https://cran.r-project.org/web/packages/gplots/index.html

8
https://cran.r-project.org/web/packages/vegan/index.html

## Supplementary material

Supplementary Figure 1 Reproducibility of the number of culturable bacteria revealed by “grain-by-grain” method: significant correlation between Experiment 1 and Experiment 2. Three sites (TM, BA, and TG) analyzed in two independent experiments.

Supplementary Figure 2 Reproducibility of the number of culturable bacteria revealed by “suspension-dilution” method: significant correlation between Experiment 1 and Experiment 2. Three sites (TM, BA, and TG) analyzed in two independent experiments.

Supplementary Figure 3 Efficiency of the “grain-by-grain” vs. “suspension-dilution” methods: significant correlation between the number of culturable bacteria using the “Grain-by-Grain” method (% N GbG/Ntot) and the number of culturable bacteria using the “suspension-dilution” method (% N SD/Ntot) in percent of the number of total bacteria (microscope observations after Syto9 staining). Three sites analyzed in two independent experiments: Timoudi (Exp1, white squares; Exp2, black squares), Béni Abbès and Taghit (blacks dots surrounded by black circle).

Supplementary Table 1 Main geographic characteristics of the three sites (TM, BA, and TG) and the nine sub-sites: TM1/TM2/TM3, BA1/BA2/BA3, and TG1/TG2/TG3.

Supplementary Table 2 Analysis of variance (ANOVA) of the number of grains per 10 mg of sand in the three sites: Timoudi (TM), Béni Abbès (BA), and Taghit (TG) measured by manual counting.

Supplementary Table 3 Main parameters of granulometer analysis, especially the mean grain diameter (in μm) calculated using the weighted volume (D[4,3]) of the sand grains in the three sites, Timoudi, Béni Abbès, and Taghit. All data were extracted from granulometer data except data followed by an asterisk.

Supplementary Table 4 Analysis of variance (ANOVA) of the number of total bacteria per grain by microscopic observations (after Syto9 staining) at the three sites: Timoudi (TM), Béni Abbès (BA), and Taghit (TG).

Supplementary Table 5A List of strains isolated in the Timoudi site: three sub-sites (TM1, TM2, and TM3), using whole 16S rRNA or V1–V4 region of 16S rRNA.

Supplementary Table 5B List of strains isolated in the Béni Abbès site: three sub-sites (BA1, BA2, and BA3), using whole 16S rRNA or V1–V4 region of 16S rRNA.

Supplementary Table 5C List of strains isolated in the Taghit site: three sub-sites (TG1, TG2, and TG3), using whole 16S rRNA or V1–V4 region of 16S rRNA.

Supplementary Table 6 List of bacterial species of the culturable microbiota at the three sites: Timoudi (TM), Béni Abbès (BA), and Taghit (TG) with the number of strains for each species.

Supplementary Table 7 Analysis of alpha diversity of the culturable microbiota at the three sites: Timoudi (TM), Béni Abbès (BA), and Taghit (TG) using Chao1, Observed_species/OTUs, and Shannon index.

Supplementary Table 8 Percentages of dominant culturable genera at the three sites: Timoudi (TM), Béni Abbès (BA), and Taghit (TG).

Supplementary Table 9 Percentage of bacterial species belonging to *Arthrobacter/* and *Massilia* genera of the culturable microbiota at the three sites: Timoudi (TM), Béni Abbès (BA), and Taghit (TG) with the number of strains for each species.

Supplementary Table 10 Dominant genera identified using 16S rRNA metabarcoding in the Timoudi (TM) site.

Supplementary Table 11 Comparison of the diversity revealed by the culture-dependent (isolated strains) and culture-independent approaches (16S rRNA metabarcoding) in the Timoudi site with in common or not genera, and their respective percentage. All genera of isolated strains are listed and only dominant genera evidenced by 16S rDNA gene metabarcoding are listed, representing 77.9% of total diversity.
