# Supplementary figures and images for: Culturing the desert microbiota
Source: Front Microbiol. 2023 Apr 11;14:1098150. doi: 10.3389/fmicb.2023.1098150 (PMC10126307; doi:10.3389/fmicb.2023.1098150)

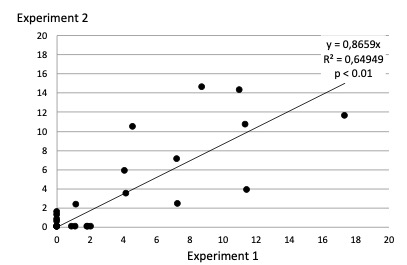

Supplement: Supplementary Figure 1 — Reproducibility of the number of culturable bacteria revealed by “grain-by-grain” method: significant correlation between Experiment 1 and Experiment 2. Three sites (TM, BA, and TG) analyzed in two independent experiments. [file Image_1.JPEG]

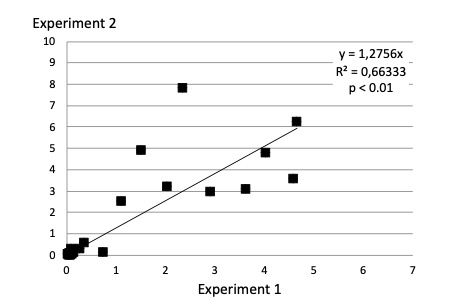

Supplement: Supplementary Figure 2 — Reproducibility of the number of culturable bacteria revealed by “suspension-dilution” method: significant correlation between Experiment 1 and Experiment 2. Three sites (TM, BA, and TG) analyzed in two independent experiments. [file Image_2.JPEG]

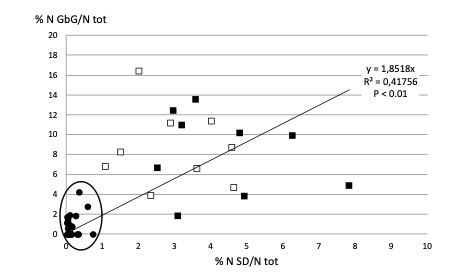

Supplement: Supplementary Figure 3 — Efficiency of the “grain-by-grain” vs. “suspension-dilution” methods: significant correlation between the number of culturable bacteria using the “Grain-by-Grain” method (% N GbG/Ntot) and the number of culturable bacteria using the “suspension-dilution” method (% N SD/Ntot) in percent of the number of total bacteria (microscope observations after Syto9 staining). Three sites analyzed in two independent experiments: Timoudi (Exp1, white squares; Exp2, black squares), Béni Abbès and Taghit (blacks dots surrounded by black circle). [file Image_3.JPEG]
