# Supplementary material for: Culturing the desert microbiota
Source: Front Microbiol. 2023 Apr 11;14:1098150. doi: 10.3389/fmicb.2023.1098150 (PMC10126307; doi:10.3389/fmicb.2023.1098150)
Supplement: Supplementary Table 1 — Main geographic characteristics of the three sites (TM, BA, and TG) and the nine sub-sites: TM1/TM2/TM3, BA1/BA2/BA3, and TG1/TG2/TG3. [file Data_Sheet_1.docx]

**Table S1:** Main geographic characteristics of the three sites (TM, BA and TG) and the 9 sub-sites: TM1/TM2/TM3, BA1/BA2/BA3 and TG1/TG2/TG3.

**Table S2:** Analysis of variance (ANOVA) of the number of grains per 10 mg of sand in the three sites: Timoudi (TM), Béni Abbès (BA) and Taghit (TG) measured by manual counting.

**Table S3:** Main parameters of granulometer analysis, especially the mean grain diameter (in µm) calculated using the weighted volume (D[4,3] of the sand grains at the three sites, Timoudi, Béni Abbès and Taghit. All data were extracted from granulometer data except data followed by an *

**Table S4:** Analysis of variance (ANOVA) of the number of total bacteria per grain by microscopic observations (after Syto9 staining) in the three sites: Timoudi (TM), Béni Abbès (BA) and Taghit (TG).

**Table S5A:** List of strains isolated in the Timoudi site: three sub-sites (TM1, TM2, TM3), using whole 16S rDNA gene or V1-V4 region of 16S rDNA gene.

**Table S5A (continued):** List of strains isolated in the Timoudi site: three sub-sites (TM1, TM2, TM3), using whole 16S rDNA gene or V1-V4 region of 16S rDNA gene.

**Table S5A (continued and final):** List of strains isolated in the Timoudi site: three sub-sites (TM1, TM2, TM3), 16S rDNA gene or V1-V4 region of 16S rDNA gene.

**Table S5B:** List of strains isolated in the Béni Abbès site: three sub-sites (BA1, BA2, BA3), using whole 16S rDNA gene or V1-V4 region of 16S rDNA gene.

**Table S5C:** List of strains isolated in the Taghit site: three sub-sites (TG1, TG2, TG3), using 16S rDNA gene or V1-V4 region of 16S rDNA gene.

**Table S6.** List of predicted bacterial species of the culturable microbiota at the three sites: Timoudi (TM), Béni Abbès (BA) and Taghit (TG) with the number of strains for each species.

**Table S7:** Analysis of alpha diversity of the culturable microbiota at the three sites: Timoudi (TM), Béni Abbès (BA) and Taghit (TG) using Chao1, Observed_species/OTUs and Shannon index.

**Table S8:** Percentages of dominant culturable predicted genera at the three sites: Timoudi (TM), Béni Abbès (BA) and Taghit (TG).

******Bacillus/Domibacillus/Lysinibacillus/Neobacillus/Novibacillus/Metabacillus/Peribacillus*

**Table S9:** Percentage of predicted bacterial species belonging to *Arthrobacter/* and *Massilia* genera of the culturable microbiota at the three sites: Timoudi (TM), Béni Abbès (BA) and Taghit (TG) with the number of strains for each species.

**Table S10**: Dominant genera predicted using 16S rDNA gene metabarcoding in the Timoudi (TM) site.

**Table S10 (Suite)**: Dominant genera predicted using 16S rDNA gene metabarcoding in the Timoudi (TM) site.

**Table S11:** Comparison of the diversity revealed by the culture-dependent (isolated strains) and culture-independent approaches (16S rDNA gene metabarcoding) in the Timoudi site with in common or not genera, and their respective percentage. All genera of isolated strains are listed and only dominant genera evidenced by 16S rDNA gene metabarcoding are listed, representing 77.9% of total diversity.
